# PD63 The Multiplier Effect Of Social Health Spending On The Brazilian Economy And Household Income

**DOI:** 10.1017/S0266462325101955

**Published:** 2025-12-29

**Authors:** Everton Macêdo Silva, Bernardo Coelho, Ivan Mardegan, Leonardo Bia, Flávia Martins

## Abstract

**Introduction:**

In Brazil, the health sector is a relevant contributor to the gross domestic product (GDP). On the other hand, the public health system can cause redistributive effects on household income. The objective of this study was to present an estimate of the multiplier effect of public health spending on GDP and household income.

**Methods:**

This analytical study was based on the national accounts for the year 2021. A social accounting matrix (SAM) was structured using values of transactions between economic sectors. This matrix was transformed into a mathematical model to capture the impact of an impulse in one sector on the others. The model consisted of complementary matrices of endogenous and exogenous accounts. The multiplier factor of the impulse was calculated, expressed as the percentage change in GDP or household income obtained by generating a 1 percent impulse in spending.

**Results:**

Based on the SAM 2021 constructed, it was possible to estimate that an additional one percent of resources applied to health by the government generated an additional 1.61 percent to the GDP. When estimated with the average multiplier effect of government consumption, a factor of 1.35 was found. Data suggested that 0.90 of this could be attributable to the additional workforce, possibly related to the demand for workers to respond to increased health services. The multiplier effect on household income demonstrated that every USD1.00 invested by the government in health generated USD1.23 in additional income for households.

**Conclusions:**

The impact of social expenditure in health on GDP and household income is positive, which is a positive contribution to the debate on increasing the allocation of resources to health. Regional differences and other social determinants are very relevant in Brazil and may be analyzed to evaluate the impact of health spending on different household settings.

